# Developing and assessing acceptability of a shared decision-making tool for selecting among HIV pre-exposure prophylaxis options in the United States of America

**DOI:** 10.1371/journal.pgph.0005557

**Published:** 2026-05-19

**Authors:** Wendy W. Davis, Andrea Mantsios, Aimee A. Metzner, Ali Talan, Jennafer Kwait, Humberto Gonzalez Rodriguez, Tahilin Sanchez Karver, Daniel Wilhite, Suyanna Barker, Angela Suarez, Ricardo Fernandez, Carlos E. Rodriguez-Diaz, Tamara Taggart, Alan Oglesby, David Wohl, Clare Barrington, Deanna Kerrigan

**Affiliations:** 1 Department of Prevention and Community Health, Milken Institute School of Public Health, George Washington University, Washington, District of Columbia, United States of America; 2 Public Health Innovation and Action (PHIA), New York, New York, United States of America; 3 ViiV Healthcare, Durham, North Carolina, United States of America; 4 Whitman-Walker Institute, Washington, District of Columbia, United States of America; 5 Department of Health Behavior, Gillings School of Global Public Health, University of North Carolina, Chapel Hill, North Carolina, United States of America; 6 La Clinica del Pueblo, Washington, District of Columbia, United States of America; 7 Division of Infectious Diseases, School of Medicine, University of North Carolina, Chapel Hill, North Carolina, United States of America; London School of Hygiene and Tropical Medicine, UNITED KINGDOM OF GREAT BRITAIN AND NORTHERN IRELAND

## Abstract

We developed a pre-exposure prophylaxis (PrEP) shared decision-making tool to support people who could benefit from PrEP in reaching their prevention goals and preferences when considering two effective PrEP modalities, oral or long-acting injectable (LAI) PrEP, in partnership with their healthcare providers (HCPs). This 2-phase study included formative research to develop a PrEP shared decision-making tool prototype followed by a pilot study to determine prototype acceptability. For the formative phase, we conducted semi-structured qualitative in-depth interviews with a diverse sample of people who could benefit from PrEP (n = 41) and HCPs (n = 20) in two community-based sites in Washington, DC, prior to and shortly after LAI PrEP became available in December 2021. Insights from in-depth interviews informed development of a PrEP shared decision-making tool prototype in both English and Spanish. This prototype was piloted in one-on-one mock clinical encounters with 37 people who could benefit from PrEP and 11 HCPs, in the two DC sites and at a university-affiliated clinic in Chapel Hill, North Carolina. After these encounters, people who could benefit from PrEP and HCPs were interviewed independently about acceptability of the prototype’s language, visuals, content and flow as well as the usability, utility, and feasibility of the prototype to support informed shared decision-making. In the formative phase, people who could benefit from PrEP had low awareness of and high interest in LAI PrEP. They and HCPs agreed that side effects and efficacy should be a primary focus of a PrEP shared decision-making tool. Participants in the mock clinical encounters found that the prototype filled information gaps, supported shared decision-making regardless of an individual’s experience with PrEP, and systematized, streamlined and normalized conversations around PrEP choice. Testing the effectiveness of the prototype in a larger-scale evaluation is highly warranted as the field seeks to address ongoing inequities in PrEP uptake.

## Introduction

Shared decision-making is defined as a collaborative process where 1) clinicians share information about treatment or prevention options, 2) a single best option is not established, and 3) clients can share information about their goals and preferences [1–5]. In shared decision-making, clinicians and clients review the best available evidence to consider trade-offs between therapeutic or prevention options, and clients select an approach that is in line with their needs and preferences [6,7]. Shared decision-making can provide information on evidence-based care options [6,7], and has been shown to improve trust in healthcare providers (HCPs) [8], medication adherence [9] and retention in preventive care [10]. Given United States (US) Food and Drug Administration (FDA) approval of long acting cabotegravir, the first FDA approved long-acting injectable (LAI) pre-exposure prophylaxis (PrEP) to prevent HIV acquisition in December 2021 [11], adding to previously approved options for a daily oral PrEP pill, shared decision-making is an optimal approach to support PrEP decision-making in selecting among two evidence-based and effective modalities for people who could benefit from PrEP [12,13].

In 2022, only 36% of the estimated 1.2 million people who could benefit from PrEP in the US were prescribed PrEP of any kind [14]. This disconnect reflects inequities in access and uptake as noted below as well as barriers at the clinic visit level. These barriers include varying degrees of comfort with prescribing PrEP [15,16] and discrepancies between relatively high provider awareness [68%; 95% confidence interval (CI) = 55–80%] and willingness to prescribe PrEP (66%; 95% CI = 54–77%), with relatively low PrEP consultations (37%; 95% CI = 25–51%) and PrEP prescriptions (24%; 95% CI = 17–32%) [16]. Factors driving this difference included a lack of knowledge and skills among providers and a lack of PrEP requests among clients. Shared decision-making holds real promise as a mechanism for stimulating PrEP consultations with all potential people who could benefit from PrEP.

Shared decision-making might also help to address disparities in PrEP access and uptake given that low awareness of PrEP can contribute to these marked disparities [14,17]. For example, among those using PrEP in the US in 2023, 92% were male and 8% were female despite the fact that 19% of new HIV diagnoses in the US that year were among women [18]. At the same time, White individuals accounted for nearly two-thirds (64%) of PrEP users while they represented one-quarter (24%) of all new HIV diagnoses. This is compared to individuals who are Black who represented 39% of all new HIV diagnoses and yet accounted for just 14% of PrEP users and Hispanic/Latine individuals who represented 31% of all new HIV diagnoses and yet accounted for just 18% of PrEP users [18]. PrEP shared decision-making might also improve uptake of LAI PrEP, which remains low, with only 1 in 200 PrEP prescriptions written for LAI PrEP [19]. Indeed, in a qualitative study of factors affecting implementation of LAI PrEP in primary care settings, a culture of shared decision-making, where people who could benefit from PrEP felt they were trusted to make the final decision about their care, was shown to be a primary facilitator of LAI PrEP implementation [20].

In thinking about PrEP choices, people who could benefit from PrEP and their HCPs have offered insights on the kinds of information that they would need for PrEP shared decision-making. For example, many studies, including a recent systematic review led by Wulandari et al., have highlighted the need for information on the effectiveness of PrEP modalities for shared decision-making [21,22]. Other studies have documented the need for information on safety [22], side effects [23,24], mode of administration [23], time and logistical challenges [23,25], and have noted that stigma and cost will also be important considerations for decision-making [23,24].

Decision support tools and decision aids have been shown to enhance shared decision-making [26], decision satisfaction [27], and medical outcomes [28], while a lack of tools has been found to limit decision-making that is led by people who could benefit from PrEP in clinical encounters [5]. Tools and aids that elicit individual perspectives, concerns, and values can be of particular value to traditionally underserved people who could benefit from PrEP as they have been shown to be protective against provider implicit bias [27]. People who could benefit from PrEP have also noted a preference for a shared decision-making tool that can be used to support one-on-one conversations with providers [29]. To our knowledge, such a support tool does not currently exist for oral and LAI PrEP. The goal of this formative study was to develop and pilot a shared decision-making tool that would support people who could benefit from PrEP and their HCPs in a conversation about PrEP and a newly available PrEP option and that would provide both people who could benefit from PrEP and HCPs with the kinds of information they identify as necessary for shared decision-making about existing and novel PrEP options.

## Methods

### Ethical considerations

All participants (HCPs and people who could benefit from PrEP) provided consent prior to each interview and were offered a $50 gift card to acknowledge time spent participating in the IDI (formative development) or the mock clinical encounter using the PrEP shared decision-making tool prototype and follow up exit interview (PrEP shared decision-making tool pilot). Study staff read and reviewed consent documents with participants in either English or Spanish in accordance with the participant’s preference. Participants provided oral consent to ensure confidentiality given that there was no identifying information collected for these one-time anonymous interviews conducted via phone or Zoom^TM^. Each participant’s consent was signed by study staff to attest that consent had been given. Study procedures were approved by George Washington University’s Institutional Review Board.

### Study design and setting

This was a study to inform the development and piloting of a shared decision-making tool for PrEP. For the formative work, we conducted semi-structured qualitative in-depth interviews (IDIs) with people who could benefit from PrEP (n = 41) and HCPs providing PrEP clinical care delivery and support services (n = 20) in two community-based clinical sites that provide HIV services in Washington, DC from 10/27/2021 to 02/25/2022 prior to and shortly after the first LAI PrEP, long-acting cabotegravir, became available. One clinic is known for its expertise in lesbian, gay, bisexual, transgender, and queer/questioning+ (LGBTQ+) care and the other is a federally qualified health center serving a low-income Latine population.

In-depth interviews informed the development of a PrEP shared decision-making tool in English and Spanish. The English and Spanish PrEP shared decision-making tool prototype was piloted in 37 mock clinical encounters with people who could benefit from PrEP and HCPs, in the two DC sites as well as at an academic-affiliated clinic in Chapel Hill, North Carolina, from 11/3/2022–3/17/2023. All three clinics provide care for clients who report or demonstrate reasons for HIV prevention. Clients at each site regularly participate in HIV related research.

### Recruitment and sample

For both phases of the study, people who could benefit from PrEP were recruited by clinic and research staff at each site in accordance with protocols that were developed jointly by co-investigators and site research staff. Inclusion criteria for people who could benefit from PrEP were that they be 18 years or older, HIV negative, and receiving care at the participating site. Inclusion criteria for HCPs were that they were affiliated with the participating site and involved in PrEP clinical care delivery and support services. On-site clinic or research staff recruited people who could benefit from PrEP by phone and by approaching potential participants at their in-person clinic visits to assess interest and availability. We sought to achieve a diverse sample in terms of PrEP use/experience (e.g., have *never* used PrEP, have *ever* used PrEP but are not using PrEP currently and *currently* using PrEP), as well as demographics such as gender, sexual orientation, and race/ethnicity. HCPs were recruited by study co-investigators at each site.

### Data collection and analysis

#### Formative development of the PrEP shared decision-making tool.

In-depth interviews were conducted in either English or Spanish using a semi-structured guide to explore knowledge and perceptions of oral and LAI PrEP. In-depth interviews were informed by Haynes et al.’s conceptual model for facilitating patient-led shared decision-making which stresses the importance of decisions that reflect an individual’s clinical state and circumstances, their preferences and relevant research evidence [30]. While clinical expertise remains centrally important in this framework, it is balanced against the provision of information and the solicitation of values and preferences. Formative in-depth interviews for this study were designed to assess awareness, knowledge and perceptions of PrEP and PrEP modalities among both people who could benefit from PrEP and HCPs. People who could benefit from PrEP were asked about any experience they might have had with PrEP, questions they would want to discuss with their HCP when choosing between oral and injectable PrEP and what kind of support tool or resource they would find helpful in PrEP shared decision-making with their HCP. Providers were asked to describe how they engaged in shared decision-making with people who could benefit from PrEP and what kind of support tool or resource would facilitate those conversations. Interviews lasted between 30 and 60 minutes and were conducted virtually by trained study-specific research staff via Zoom™ (San Jose, CA) from a private location of the participant’s choosing. All IDIs were audio recorded with the consent of participants. Interviewers completed field notes using a template after each interview to document interview dynamics and salient themes. Audio recordings of interviews were transcribed in the language in which the interview was conducted.

Drawing on key domains from the interview guides and field notes, codebooks were developed for both people who could benefit from PrEP and HCP interviews including code names, definitions, and instructions for use. Codes were applied to the transcribed interviews using the ATLAS.ti (version 1.0.50; Scientific Software Development GmbH, Eden Prairie, MN) qualitative data analysis software. Memos were written throughout the coding process to document emerging themes and connections, and codebooks were modified as needed during the coding process [31]. Key themes generated from the synthesis of code output from both people who could benefit from PrEP and HCP interviews were incorporated into a cross interview analytic matrix that provided the foundation for the creation of a PrEP shared decision-making tool prototype, guiding the specific topics, domains, language and visuals that were included (see Table 2).

The PrEP prototype was two sided with the first side providing foundational information about oral and injectable PrEP including defining how each are used as well as providing information on effectiveness and side effects. The second side focused on personal preference and values associated with the mechanics, (e.g., swallowing a pill or receiving an injection); convenience (e.g., remembering to take a pill every day or getting to a clinic every two months) and potential stigma and discrimination (e.g., worrying more about others discovering your pills or being seen at a clinic waiting to get an injection) associated with both PrEP modalities. At the bottom of the second side, the PrEP shared decision-making tool prototype suggested that people who could benefit from PrEP could now be ready for a conversation with the HCP about PrEP preferences and needs and listed a series of specific topics that people who could benefit from PrEP might also want to discuss with the HCP. Topics included: missed doses; interactions with other medicines; getting started with PrEP; cost and coverage; reproductive health considerations; protection against other STIs; other HIV prevention options, etc. Once the prototype was finalized, it was translated into Spanish.

#### PrEP shared decision-making tool pilot.

The PrEP shared decision-making tool prototype was piloted in paired one-on-one simulated or mock clinical encounters with an HCP and a person who could benefit from PrEP using either the English or Spanish PrEP shared decision-making tool prototype based on the language preference of the person who could benefit from PrEP. The intent of the mock clinical encounter was for people who could benefit from PrEP and HCPs to review the PrEP shared decision-making tool prototype together, as would occur in an actual clinical appointment, and for HCPs to respond to any questions it raised for people who could benefit from PrEP, again as would occur in an actual clinical appointment. Mock clinical encounters lasted for approximately 30 minutes during which the HCP and the person who could benefit from PrEP proceeded through the content provided on the first side and then the second side of the PrEP shared decision-making tool prototype. The mock clinical encounter concluded with an opportunity for HCP and people who could benefit from PrEP participants to have a conversation about preferences and needs as well as to discuss any of the specific topics noted above. In advance of the mock clinical encounter, a member of our study team met briefly with HCP participants and reviewed with them both the intent and flow of the PrEP shared decision-making tool prototype as well as an HCP PrEP information resource that our team had previously developed. The HCP PrEP information resource provided details on oral and LAI PrEP clinical trials in different populations as well as processes for starting, switching and monitoring PrEP use and medication interactions that were not included in the PrEP shared decision-making tool and that could aid in answering specific questions that people who could benefit from PrEP might have about oral and LAI PrEP as they consider PrEP modality choices. HCPs were also prepared to offer information on how to access PrEP within their clinic if requested by the person who could benefit from PrEP study participant. Mock clinical encounters were conducted either in person with a paper version of the PrEP shared decision-making tool or virtually via Zoom™ with an electronic version of the PrEP shared decision-making tool in response to participant preference or need.

After each mock clinical encounter we conducted a semi-structured qualitative exit interview with each participant (person who could benefit from PrEP and HCP) independently and solicited feedback, using open ended questions, on the language, visuals, content, and flow of the PrEP shared decision-making tool prototype as well as on changes that might be made for improving the PrEP shared decision-making tool prototype including if there were any gaps in the material covered by the PrEP shared decision-making tool prototype. We asked participants to share their perspectives on the acceptability, usability, utility, and feasibility of the PrEP shared decision-making tool prototype in terms of facilitating informed shared decision-making as well as their perceptions of the mock clinical encounter. People who could benefit from PrEP were asked to share how they felt before and during the mock clinical encounter and HCPs were asked to share their impressions of how people who could benefit from PrEP approached and experienced the mock clinical encounter. Healthcare providers were also asked about the HCP PrEP information resource including if it helped them prepare for the mock clinical encounter and was useful during the encounter. In some instances, if an HCP had conducted more than one encounter, they were interviewed about multiple encounters in a single exit interview allowing for a comparison of the utility of the PrEP shared decision-making tool prototype for different people who could benefit from PrEP and potentially across different populations. Exit interviews were conducted in English or Spanish and lasted for between 30 and 45 minutes. Exit interviews were conducted virtually by trained study-specific research staff via phone or Zoom™ from a private location of the participant’s choosing and were audio recorded with the consent of participants.

Interviewers completed a template after each qualitative exit interview cataloguing their field notes on the topics discussed in the exit interview. Template responses were compiled and synthesized into cross-interview analytic matrices and then compared across sites and from both people who could benefit from PrEP and HCP perspectives. Synthesized findings were used to identify areas of consensus for content and format refinement of the PrEP shared decision-making tool prototype as well as considerations for implementation. These areas of consensus formed the foundation for recommended revisions to improve the clarity and utility of the PrEP shared decision-making tool prototype.

## Results

### Formative development of the PrEP shared decision-making tool

#### Sample characteristics.

People who could benefit from PrEP who participated in the formative IDIs were diverse in terms of their experience with PrEP and their self-reported demographic characteristics (Table 1). All 20 HCPs included in the formative IDIs provided PrEP clinical care delivery and support services; 55% were PrEP prescribers. Healthcare providers served in a range of roles from health educator and program specialist to nurse practitioner and physician. Ninety-five percent of HCPs reported HIV as an area of specialty, many HCPs had expertise in other domains as well including general practice and primary care and family planning and obstetrics and gynecology.

**Table 1:** Formative IDI participant characteristics.

People who could benefit from PrEP (N = 41)	n	%
PrEP use		
Current	17	41
Ever	12	29
Never	12	29
Gender		
Cisgender male	16	39
Cisgender female	12	29
Non-binary	1	2
Transgender female	12	29
Age in years	Median (range)	39 (23-73)
Sexual orientation		
Bisexual	7	17
Heterosexual	16	39
Gay/lesbian	13	32
Pansexual	1	2
Queer	2	5
Prefer not to say	2	5
Race/ethnicity		
African American/Black	15	37
Asian	1	2
Latine	21	51
Multiple races	1	2
White	3	7
**HCPs (N = 20)**	n	%
Provider type		
Doctor of Osteopathic Medicine	1	5
Health educator	2	10
Medical assistant	1	5
Nurse	2	10
Nurse practitioner	3	15
Physician	3	15
Physician Assistant/Associate	4	20
PrEP case manager	1	5
PrEP program specialist	3	15
Specialty^a^		
General practice/primary care	12	60
Family planning	11	55
HIV	19	95
OB/GYN	11	55
Sexually transmitted infections	15	75
PrEP involvement		
Service delivery	20	100
Able to prescribe	11	55

HCP, healthcare provider; IDI, in-depth interview; OB/GYN, obstetrics and gynecology; PrEP, pre-exposure prophylaxis.

^a^HCPs could list more than 1 specialty.

#### Awareness of oral and LAI PrEP.

Most people who could benefit from PrEP (n = 33, 80%) were unaware of LAI PrEP as an option. Three cisgender female people who could benefit from PrEP were not aware of PrEP at all. The majority of people who could benefit from PrEP who had heard of PrEP learned about it through their clinic or HCP. In a few instances the conversation with their HCP was prompted in part by the individual having a partner that was living with HIV. Other individuals noted that they had heard of PrEP via friends or through media outlets.

Several people who could benefit from PrEP noted the important role that HCPs played in awareness and uptake of PrEP. A person who could benefit from PrEP who had not heard of PrEP before the IDI explained that she had had a conversation about the risks of HIV with her doctor but that her doctor had not told her about PrEP:

*No, he didn’t talk to me about pills. They tested me, they took my blood and tested me… but they didn’t tell me about pills or anything…they only gave a bag (of condoms) to me.* (Heterosexual Latina cisgender female, never PrEP user, age 56)

Another person who could benefit from PrEP had heard of PrEP through TV commercials but explained that they would want to discuss it with their HCP before pursuing:

*I mean, you see it on the commercials and stuff. But I didn’t never know what it was called. I used to just see it on the commercials all the time. You know they have a commercial that come on and you don’t really pay no attention…I see it on TV but I don’t really, like I say, I would have to ask my doctor about something like that, you know what I mean?* (Heterosexual Black cisgender male, never PrEP user, age 57)

This participant also noted that he did not recall his doctor ever mentioning PrEP to him. One person who could benefit from PrEP who was aware of LAI PrEP and had heard of it being approved by the FDA was discouraged from trying it by their HCP who felt that there was not enough information about it:

*I just heard on the news that there’s, like, a new injectable that you only have to take like once a month and I, so I contacted my doctor like maybe a week ago-- a week or two ago just like saying, “Hey, like, can I take this injectable? They said it may be more effective.” They told me that it wasn’t actually that effective and, like, there’s not that much information about it yet and that I don’t need it because, like, the pill is still better.* (Lesbian Black transgender woman, current PrEP user, age 30)

Of the 20 HCPs interviewed, 15 were aware of LAI PrEP although three of these noted that their knowledge of LAI PrEP was limited. Healthcare providers had learned about LAI PrEP either through clinic programming or on the internet.

#### Initial perceptions of LAI PrEP.

Almost all people who could benefit from PrEP at both sites expressed interest in learning more about LAI PrEP and the majority expressed openness to trying LAI PrEP. A few people who could benefit from PrEP in each site were confident that they would prefer LAI PrEP over daily oral PrEP while others suggested that once they had a full understanding of LAI PrEP they would likely choose it. At each site, there were also a few people who could benefit from PrEP who were certain that they would not want to try LAI PrEP.

About half of all people who could benefit from PrEP with a positive initial perception of LAI PrEP noted challenges with remembering to take a daily pill. As one participant explained:

*And that’s what bothered me the most because sometimes you forget, so I said, I forgot to take the pill. So I had to be very aware of my pill, I didn’t like that part very much because I had to watch it every day, if you stopped taking it they said it wasn’t as effective.* (Bisexual Latino cisgender male, prior PrEP user, age 34)

People who could benefit from PrEP also observed that LAI PrEP would be especially valuable for people who could benefit from PrEP who were traveling as they would then not need to remember their pills.

Some people who could benefit from PrEP noted that they liked the idea of LAI PrEP going straight into their bloodstream, perceiving this as “instant protection,” while others were concerned about whether the effectiveness of LAI PrEP would ebb and flow. A few people who could benefit from PrEP explained that a fear of needles or a concern about injection site pain meant that they would not be interested in trying LAI PrEP while others noted that the fact that they were currently on an injection regimen for another medication would lead them to consider LAI PrEP. In some instances, people who could benefit from PrEP found the need to go to a clinic every 2 months for LAI drawback, noting challenges with scheduling appointments, work schedules, transportation difficulties, and childcare needs. A couple of people who could benefit from PrEP mentioned a concern about circumstances in which clinics closed as happened during the COVID-19 pandemic and what that might mean if they were scheduled for an LAI PrEP injection.

Healthcare providers felt that LAI PrEP could be an appealing option for people who could benefit from PrEP, especially those that might struggle with adherence.

*I mean, it’s a lot better to know someone’s adherence if you’re giving them the shot. So I think I have a lot more confidence in it, just knowing that it was actually given in our clinic. I mean, I don’t even have confidence that people will go pick up the PrEP, let alone take it how they’re supposed to take it.* (cisgender female, Physician Assistant)

Healthcare providers also felt that people who could benefit from PrEP who experience instability in their daily lives such as individuals who are unstably housed or struggle to meet basic needs, individuals who use substances, sex workers, individuals experiencing domestic violence, younger individuals who might have a less regular schedule, and individuals who find remembering to take a pill every day a challenge could benefit from LAI PrEP. Healthcare providers also noted that those who are concerned about privacy and stigma related to oral PrEP use could benefit from LAI PrEP and that LAI PrEP would remove the emotional burden of thinking about HIV risk every day.

Healthcare providers had concerns about the logistics of implementing LAI PrEP including being short-staffed and the perceived mismatch between the LAI PrEP injection schedule (every 2 months) and the recommended timing of PrEP testing guidelines (every 3 months), potential issues with cost and insurance coverage, and schedule and transportation constraints to clinic access and retention in care.

*The cons that I’m thinking of when it comes to [our] patient population are… whether they can get to [the clinic] every 2 months; whether it’s conflicts with work; whether it’s transportation issues; whether it’s childcare issues… And obviously I understand it’s a, you know, personal option to choose the long-acting, but I think also if you have, we have patients that are kind of in and out of care and so that might not be a good option for them. So just kind of thinking of some of the maybe the social determinants of health that would prevent this from being a good option for some individuals.* (cisgender female, Case Manager)

#### Domains that emerged as important to include in a PrEP shared decision-making tool.

People who could benefit from PrEP had many suggestions for topics that should be included in a PrEP shared decision-making tool. More than three quarters of people who could benefit from PrEP shared a desire to have information on both short- and long-term side effects. As one person who could benefit from PrEP described:

*Explain to me that the injectable is safe for my body, because I already know everything about the oral one, but I would like you to tell me in that form that it is safe and that it will not affect me in the future. Side effects, I would like to know what effects it has, if that injectable has different side effects than the oral one.* (Lesbian Latina transgender female, prior but not current PrEP user, age 41)

More than half of people who could benefit from PrEP also expressed a desire to have information on the effectiveness of each modality, with some suggesting that percentages be included. Another important topic for many was to have information on the impact of either daily oral or LAI PrEP on their existing medical conditions and/or other medications that they might be taking including, in some instances, hormones. Several people who could benefit from PrEP also wanted to have information on the mechanics of receiving LAI PrEP including where in the body it is injected and what they would feel after an injection. A few people who could benefit from PrEP wanted information on cost and insurance coverage to be included in the PrEP shared decision-making tool while others wanted to know about the level of protection from LAI PrEP over the 2-month period between injections. Some people who could benefit from PrEP would want the PrEP shared decision-making tool to touch on the potential impact of either PrEP modality on fertility and also on what the process would be to transition from daily oral to LAI PrEP.

Like people who could benefit from PrEP, the majority of HCPs wanted information on short- and long-term side effects included on a PrEP shared decision-making tool. Many HCPs also wanted to see information on effectiveness including real-world effectiveness:

*And then also talking about the, like, basically talking about effectiveness at preventing HIV. So writing next to the pills that if you take the pill every day, it’s this effective. And this is the real-world efficacy. Because I feel like that is important to know, like…if you take it every day versus what the real-world efficacy is.* (genderqueer Physician Associate)

Many HCPs thought it would be important to talk with people who could benefit from PrEP about their ability to adhere to either modality and to explain the implications of missed doses. Healthcare providers also thought that the PrEP shared decision-making tool should reference the cost and insurance coverage of each modality. Healthcare providers felt that it would be essential to make sure that people who could benefit from PrEP understood exactly what PrEP is, have a visual image that demonstrates the size of the pill and the injection needle, and have an understanding of the administration and timing of each modality. Healthcare providers suggested that a PrEP shared decision-making tool acknowledge fear of needles, include information on interactions with other medicines, address whether each modality can be used in pregnancy or when breastfeeding and make clear that PrEP does not protect against other sexually transmitted infections (STIs). Healthcare providers observed that it would be important to explain what is involved in switching between modalities, time to effectiveness of each modality, and whether LAI PrEP effectiveness wanes over time. Several HCPs mentioned that the PrEP shared decision-making tool should touch on issues of stigma and privacy tied to PrEP use. Finally, many HCPs suggested that the shared decision-making tool present information in a way that facilitated a side-by-side comparison.

Based on feedback from people who could benefit from PrEP and HCP participants, we developed an outline of how key discussion topics could be organized and presented in a PrEP shared decision-making tool (Table 2). People who could benefit from PrEP and HCPs also offered many suggestions for how a PrEP shared decision-making tool could be formatted and implemented as noted in Table 3.

**Table 2:** PrEP shared decision-making tool suggested topics and domains.

Domain	Definition	
PrEP overview	What is PrEP overall and how does it work to prevent HIV infection?	
Domain	LAI PrEP	Oral PrEP
Forms of PrEP	Show relative needle size and injection location	Show relative size and shape of a single pill
Frequency	Show 1 injection appointment on the calendar for a period of 2 months	Show 1 pill every day on the calendar over the course of 2 months
Logistics	Show in clinic given by provider	Show in home taken by oneself
Side effects	List most common side effects	List most common side effects
Effectiveness	Show percent protection	Show percent protection
Mechanics	Would you prefer to receive an injection?	Would you prefer to swallow a pill?
Convenience	How easy would it be to get to a clinic appointment every 2 months?	How easy would it be to take a pill every day/integrate it into daily life?
Stigma and discrimination	Would you worry about being seen at or going to clinic?	Would it worry you if others saw your pills?
**Other topics**		
Starting, stopping, and switching oral and LAI PrEP		
Missed pills, missed appointments		
Protection from other STIs		
Interactions with other medicines		
Financial/insurance status and coverage; access to financial assistance?		
Population dynamics: cisgender women (e.g., fertility, pregnancy, breastfeeding, partner considerations; transgender women (e.g., hormones)		

LAI, long-acting injectable; PrEP, pre-exposure prophylaxis; STI, sexually transmitted infection.

**Table 3:** People who could benefit from PrEP and HCP suggestions for the format and implementation of a PrEP shared decision-making tool.

Format	
Potential modalities	Printed: pamphlets, flyers, info cards (with QR code)
	Electronic: website, phone app, tablet, electronic medical record
	Social media: TikTok
Appearance	Less text
	More visuals
	Engaging colors
	Infographics, graphs and charts
	Decision trees
**Implementation**	
Access to PrEP shared decision-making tool	People who could benefit from PrEP have access to the shared decision-making tool before and after clinic visit
	Have PrEP shared decision-making tool available in clinic waiting rooms
HCP support	Training such as PowerPoint presentations, webinars, in-service trainings
Clinic preparation	Anticipate staffing demands, scheduling logistics
	Support from PrEP navigators, health education specialists, insurance navigators, and public benefits staff

HCP, healthcare provider; PrEP, pre-exposure prophylaxis.

### PrEP shared decision-making tool pilot

#### Sample characteristics.

Of the 37 people who could benefit from PrEP who participated in the mock clinical encounters in which the PrEP shared decision-making tool was piloted (Table 4), a majority (62%) had never used PrEP, with 38% reporting ever having used PrEP. People who could benefit from PrEP represented diversity in gender, sexual orientation, and race and ethnicity. Healthcare providers who participated in the mock clinical encounters in which the PrEP shared decision-making tool prototype was piloted were all involved in PrEP delivery and support and represented a range of provider types (Table 4).

**Table 4:** PrEP shared decision-making tool pilot: participant characteristics.

People who could benefit from PrEP (N = 37)	n	%
PrEP use, ever		
No	23	62
Yes	14	38
Gender		
Cisgender female	6	16
Cisgender male	22	59
Non-binary	5	13
Non-identifying	1	3
Transgender female	2	5
Transgender male	1	3
Age in years	Median (range)	47 (29-77)
Sexual orientation		
Bisexual	7	19
Bisexual/Pansexual	1	3
Gay/Lesbian	15	41
Heterosexual	8	22
Pansexual	1	3
Queer	1	3
Prefer not to say	4	11
Race/ethnicity		
African American/Black	8	22
Asian	2	5
Latine	10	27
White	17	46
**HCPs (N = 11)**	n	
Provider type		
Physician	6	55
Physician Assistant/Associate	4	36
PrEP Program Specialist	1	9

HCP, healthcare provider; PrEP, pre-exposure prophylaxis.

#### PrEP shared decision-making tool filled information gaps and supported decision-making.

Both people who could benefit from PrEP and HCPs who participated in the mock clinical encounters with the PrEP shared decision-making tool prototype found its language; visuals; content including information on oral and injectable PrEP use, effectiveness and side effects, and personal preference topics including pill versus injection, managing pills at home versus considerations with visiting a clinic for injections and additional specific topics as noted above; and flow from information included on the first side to preference topics covered on the second side to be acceptable. They noted that it filled information gaps and supported the decision-making process. Specific suggestions for improvement are noted below. Among people who could benefit from PrEP, the prototype worked well for individuals who had prior PrEP knowledge or experience as well as for those who had no prior PrEP knowledge or experience:

*I’m thinking this would have been nice when I started-- when I talked to the doctor about going on PrEP because he really didn’t discuss much. It was like I want to go on PrEP. Okay. You got to test every three months, I think is what he told me. And here’s your prescription, goodbye. But this, you know, I don’t even think he told me about side effects. This goes over that this. … And I think having this guide was a good communication tool. It opened up communications.* (Gay White cisgender male, current PrEP user, age 65)

*It’s (PrEP) something I’ve heard about before. This was like a much better explanation than the explanations my friends have given me. Everything about it [the PrEP shared decision-making tool prototype] was good, it was clear and everything, and very helpful, actually.* (Bisexual Latino cisgender male, never PrEP user, age 32)

The PrEP shared decision-making tool prototype was found to support decision-making about PrEP use overall and around appropriate PrEP type:

*If anything, it moved me closer to taking it…I don’t know if having the two choices is making me think about it more… but…having the discussion that I had with the provider kind of helped me to focus on the important stuff.* (Bisexual Black cisgender male, never PrEP user, age 74)

*[The PrEP shared decision-making tool prototype] was actually quite positive, in the sense that I learned something new, because I always thought, like, PrEP was just a pill, which is fine. But I thought that it was kind of annoying, every day having to take one, or waiting to take it. But I didn’t know that, it was something new for me, to learn that now here is the injection and that your injection can last like two months…it [LAI PrEP] was more interesting, a much better option then to have to be taking the pill. I don’t know how long it has been, but I had never realized that it was an injection, which for me would be much more preferable than the pill.* (Bisexual Latino cisgender male, never PrEP user, age 32)

For current PrEP users, the PrEP shared decision-making tool prototype either solidified the decision to stay on oral PrEP or encouraged trying LAI PrEP. Several individuals who reported being pleased with their current oral PrEP use described that the PrEP shared decision-making tool moved them toward wanting to switch to LAI PrEP, noting that the shared decision-making tool provided a lot more information than they previously had about this option. One individual who currently used oral PrEP on demand described how he had heard about the possibility of an injectable option and it appealed to him so he was interested to learn more going into the conversation. He described the PrEP shared decision-making tool prototype as particularly helpful *“because what it says in the brochure is also explained to one personally and what it says is good. One understands perfectly”* (Heterosexual Latino cisgender male, current PrEP user, age 43). Regardless of PrEP use history, most people who could benefit from PrEP indicated that they left the mock clinical encounter knowing more about PrEP overall than they had previously.

#### PrEP shared decision-making tool systematizes PrEP conversations.

Both people who could benefit from PrEP and HCPs felt that the PrEP shared decision-making tool prototype systematized the mock clinical conversation keeping both parties focused on the reason for the visit and walking them through a dialogue that ensured that key topics were comprehensively addressed and organized in a way that supported a productive shared decision-making conversation.

*I think the value of the tool is to have a ready-made kind of discussion, a template…the complete visit was on that tool, and if you had covered all of that you probably got all the information you could in that timeframe.”* (cisgender female Physician Associate)

*What I liked about it was it was structured and allowed for a flow that made sense clinically.* (cisgender male Physician)

People who could benefit from PrEP described the flow of the prototype as clear and liked that it provided a step-wise list of topics to cover with the HCP that “made sense” and was “logical.”

*For me, the linear description that the tool provides was helpful. You know, it’s this, then it’s that, then it’s this. You know, you’re making the decision of a pill every day or a clinic visit. That worked. That kind of linear, progression works for me.* (Gay White cisgender male, never PrEP user, age 71)

Referring to the flow and the coverage of important topics around the two PrEP options that the PrEP shared decision-making tool prototype provided, one person who could benefit from PrEP simply stated:

*“I loved it. It was very much, ‘Who, what, when, where, how?’”* (Gay Black cisgender male, current PrEP user, age 35)

#### PrEP shared decision-making tool normalizes conversations around PrEP.

The format of the PrEP shared decision-making tool prototype and the language used were both cited by people who could benefit from PrEP as helping to normalize the conversation around PrEP. People who could benefit from PrEP reported that being provided with information on two modalities of PrEP felt neutral and the format made for an interactive session. People who could benefit from PrEP felt the PrEP shared decision-making tool promoted a back-and-forth, so they were not being *“talked at”* but rather engaged in a dialogue. People who could benefit from PrEP felt that the PrEP shared decision-making tool prototype was like a *“third party in the room”* saying it served as *“an icebreaker”* that helped them have a conversation with their HCP about PrEP.

People who could benefit from PrEP across the sites noted that the shared decision-making tool language was *“conversational”* and *“easy to understand”* and that this facilitated a smooth dialogue focused on the decision-making process. Furthermore, the language was described as simple, concise, *“self-explanatory,”* and *“not overly medical”* which made the PrEP shared decision-making tool and the conversation more approachable and comfortable. Participants connected the fact that the shared decision-making tool did not use medical jargon or feel overly medical with the idea that it *“leveled the playing field”* for conversations about PrEP. The final section of the PrEP shared decision-making tool, which lists specific topics for further discussion, helped people who could benefit from PrEP feel that they didn’t have to raise potentially uncomfortable topics and suggested that these are *“normal things to wonder about.”*

A prevalent sentiment among people who could benefit from PrEP involved in the pilot’s mock clinical encounters was that the PrEP shared decision-making tool prototype normalized a conversation that had the potential to be stigmatizing. Because the PrEP shared decision-making tool prototype does not include a risk assessment, individuals felt they did not have to justify their interest in PrEP use. People who could benefit from PrEP overwhelmingly reported liking that they did not have to talk about risk behaviors and reported that this, along with *“non-judgmental language,”* helped the conversation feel more focused on PrEP rather than on them and their behaviors. This was particularly salient for subgroups who felt that they are not considered as candidates for PrEP such as older populations.

For Spanish-speaking people who could benefit from PrEP, conversations about PrEP were further eased by having the shared decision-making tool in Spanish. Latine participants in the sample described it as *“a relief”* and *“empowering.”*

*I liked it a lot because it is in Spanish, I was able to read it too and understand it very well, [the provider] explained it to me well.* (Bisexual Latino cisgender male, never PrEP user, age 45)

Latinas in the sample who had low literacy and self-described low health literacy discussed how having material like this was helpful.

*“The [shared decision-making tool] helped me have a more detailed conversation than my previous conversations with [my provider] about PrEP.”* (Heterosexual Latina cisgender female, never PrEP user, age 54)

#### Suggestions for improving PrEP shared decision-making tool content.

**Fig 1:**
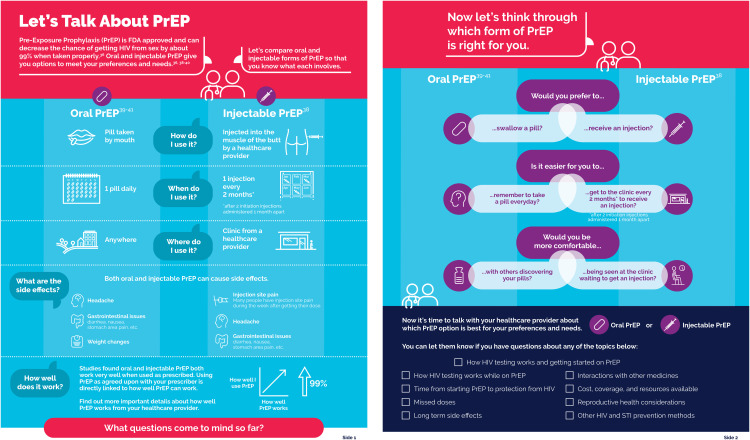
Pre-exposure prophylaxis shared decision-making tool thumbnail (English) [32–35].

**Fig 2:**
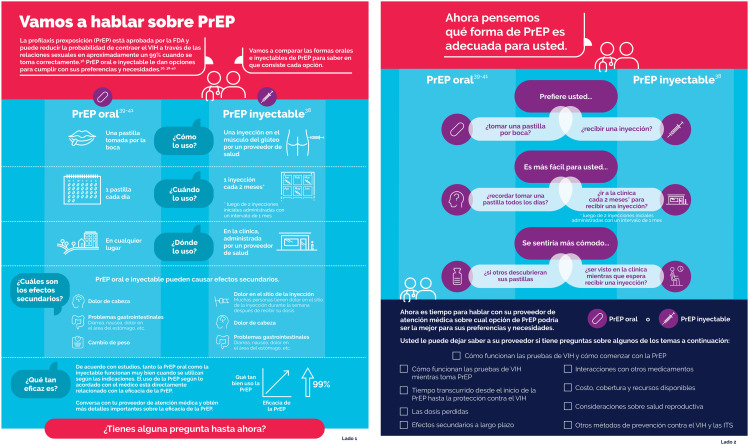
Pre-exposure prophylaxis shared decision-making tool thumbnail (Spanish) [32–35].

People who could benefit from PrEP and HCPs provided useful feedback on how to improve the clarity and usability of the PrEP shared decision-making tool prototype. For example, both HCPs and people who could benefit from PrEP found that the text on side effects needed greater clarity and that the text about effectiveness was vague and could be misinterpreted to suggest that LAI PrEP was more effective than oral PrEP. Healthcare providers and people who could benefit from PrEP also felt that a decision tree within the PrEP shared decision-making tool prototype forced a choice that was not necessarily binary, suggested stigma when some individuals might not experience stigma, and neglected to acknowledge the anxiety and stress that some people who could benefit from PrEP might feel about either PrEP modality. Other suggestions from both people who could benefit from PrEP and HCPs added to the clarity and comprehensiveness of the PrEP shared decision-making tool prototype. After integrating these suggestions, a final set of English and Spanish PrEP shared decision-making tools were developed and are shown in Fig 1 and 2 as thumbnails and are available in full page view here.

#### Feedback on an HCP PrEP information resource.

Healthcare providers also shared feedback on the HCP PrEP information resource that was shared with participating HCPs prior to the mock clinical encounters. In particular, they noted that it would be helpful for an HCP information resource such as the one they saw to provide: information on how to start PrEP and switch modalities; to explain, as data availability allows, the time to efficacy for each modality; to clarify that oral PrEP needs to be taken at the same time each day, and that there is a potential for resistance associated mutations when HIV is acquired while on PrEP. Health care providers also suggested that a provider information resource include data on long-term side effects, for example related to bone density and kidney functioning, as well as on how long short-term side effects last. They observed that a HCP PrEP information resource should provide details on potential interactions with blood pressure and diabetes medications and substance use. They also felt that in discussions of PrEP use in different populations an information resource should include the brand names of PrEP drugs and which populations they had been approved for as well as provide detail and considerations for transgender population and people who inject drugs.

#### Considerations when implementing the PrEP shared decision-making tool.

People who could benefit from PrEP and HCPs participating in the exit interviews also offered insights on implementation of the PrEP shared decision-making tool, which are outlined in Table 5. These included suggestions on activities that both people who could benefit from PrEP and HCPs could engage in, in advance of a clinic visit, and considerations and resources needed during review of the PrEP shared decision-making tool.

**Table 5:** PrEP shared decision-making tool implementation suggestions.

Timing	Feedback	Source
Pre-shared decision-making tool use	Share tool ahead of time; allows people who could benefit from PrEP to be prepared for conversation with clear expectations and leads to focused conversations	People who could benefit from PrEP
	Provide training on PrEP shared decision-making tool and resources to answer questions	HCPs
During clinic visit with PrEP shared decision-making tool	Provider dynamics and rapport matter • Trust with HCP necessary for many people who could benefit from PrEP (e.g., Latine populations) • Some people who could benefit from PrEP preferred to speak with HCPs with a high degree of PrEP expertise or experience working with specific groups (e.g., LGBTQ+), others were comfortable with their primary care provider	People who could benefit from PrEP
	Make a digital format available	People who could benefit from PrEP
	Virtual encounters work for reviewing/discussing the tool	People who could benefit from PrEP/ HCPs
	Avoid heteronormative language in discussing PrEP study findings	People who could benefit from PrEP
	Accessibility • Spanish language shared decision-making tool empowered people who could benefit from PrEP for dialogue • Be able to provide clinical trial information in accessible language	People who could benefit from PrEP
	Additional information needed on: • Cost and insurance coverage • How to access LAI PrEP	People who could benefit from PrEP/ HCPs

HCP, healthcare provider; LAI, long-acting injectable; PrEP, pre-exposure prophylaxis.

In addition to the suggestions listed in Table 5 related to use of the PrEP shared decision-making tool during clinic visits, people who could benefit from PrEP also observed that the PrEP shared decision-making tool could have value as an educational tool more broadly and that community talks, including “charlas” or small group education, could be another mechanism for raising awareness of PrEP and PrEP options. Healthcare providers felt that the PrEP shared decision-making tool could have utility in primary care and obstetrics and gynecology clinical visits.

## Discussion

This formative study to develop and pilot a PrEP shared decision-making tool was conducted among a diverse group of people who could benefit from PrEP with a range of experience with and knowledge of PrEP and a diverse group of HCPs serving in a range of clinical roles related to the delivery and support of PrEP. Awareness of LAI PrEP was low across people who could benefit from PrEP, echoing previously documented and ongoing low awareness of LAI PrEP in US [36–39] and international settings [40–44]. Despite low awareness of LAI PrEP, a majority of people who could benefit from PrEP in both DC settings expressed an interest in and openness to trying LAI PrEP. This reflects a growing body of literature which suggests that there is high interest in LAI PrEP across populations and settings [25,45–50].

Findings from the formative phase of this research echoed previous research on shared decision-making and PrEP which have found that side effects [24,25,49,51–54], effectiveness [21,25,49,52,54] and the ease of taking a pill every day or receiving an injection every 2 months are high-priority topics [21,25]. In mock clinical encounters with the PrEP shared decision-making tool prototype, people who could benefit from PrEP responded well to its emphasis on efficacy and side effects and its inclusion of the physical and emotional ease of taking pills or receiving injections as a decision point.

Participants in the formative phase of this research also stressed the importance of minimizing text and incorporating visuals as a way of making information easier to understand and more digestible, especially for those with lower literacy levels. Prior research on the use of visual aids in materials to promote the prevention and detection of STIs have found graphics are a highly effective, transparent, and memorable means for communicating key topics [55]. People who could benefit from PrEP and HCPs piloting the PrEP shared decision-making tool prototype in mock clinical encounters had overwhelmingly positive responses to the use of visuals throughout the PrEP shared decision-making tool, reporting that they made it more accessible for people who could benefit from PrEP, helped both parties follow along with the content, and broke up the text so as to not be too visually dense.

In developing the PrEP shared decision-making tool prototype, our team worked to incorporate language that was the right balance of comprehensive and comprehensible. Research has shown that while providers may assume explanations are understood, misunderstandings may occur due to low reading comprehension skills and that these are associated with poor health outcomes [56]. People who could benefit from PrEP using the PrEP shared decision-making tool prototype in mock clinical encounters praised the approachable language it used which was described as neither overly medical nor “dumbed down,” striking a balance that the majority of participants reported felt engaging and empowering. Prior research has also demonstrated that language barriers have adverse impacts on healthcare delivery and health outcomes for Latine populations [57]. Several HCPs interviewed for the formative development of the PrEP shared decision-making tool prototype noted the potential barrier that neglecting to provide the prototype in an individual’s native language could present. The Spanish version of the PrEP shared decision-making tool prototype was well received by Spanish-speaking people who could benefit from PrEP who expressed relief and reported feeling grateful to have the information in their native language. This was particularly important given low literacy and low health literacy among this sub-sample.

This research supports the overall benefits for people who could benefit from PrEP and HCPs of a shared decision-making tool to discuss and weigh oral and LAI PrEP options and demonstrates that the PrEP shared decision-making tool prototype developed through this research was effective in providing support to both parties in the PrEP decision-making process. In this research, the existence of a PrEP shared decision-making tool allowed people who could benefit from PrEP to move from no or low awareness of LAI PrEP to demonstrating a clear preference for a PrEP modality once they had viewed the PrEP shared decision-making tool prototype. Current PrEP users also found that the PrEP shared decision-making tool prototype addressed knowledge gaps about oral PrEP.

Findings from this research also advance current understanding by showing how a PrEP shared decision-making tool can serve to systematize and normalize conversations between people who could benefit from PrEP and HCPs. Healthcare providers were satisfied that the PrEP shared decision-making tool provided them with the content they needed to cover in a format that was easy for them to use and that facilitated a systematic delivery of information on both PrEP modalities. People who could benefit from PrEP were pleased that the PrEP shared decision-making tool seemed to be normalizing the PrEP conversation in a way that left them feeling like they were not being judged but rather engaging in a medical decision-making process with their HCP. People who could benefit from PrEP also noticed and appreciated that there was no risk assessment included in the PrEP shared decision-making tool and thus, they never had to disclose information to the HCP about partners or behaviors, which they reported can lead to feelings of being judged. Studies have documented discomfort with disclosing risk behavior or gender identity to HCPs due to fear of being judged or treated harshly [58–60], which may limit access to care and contribute to inequities in PrEP uptake [60,61].

Study limitations include the potential that one of the participating clinics might have a higher-than-average awareness of and training in PrEP options and care provision. Study results may also not be applicable for all people who could benefit from PrEP including people who inject drugs given participant demographics in the study sites included in the formative and pilot phases of this research. Our evaluation of the acceptability and feasibility of the PrEP shared decision-making tool is based on mock clinical encounters which may not fully identify challenges that can emerge in an actual clinic visit and may be subject to a social desirability bias.

Findings from this research suggest the need for additional activities and research to optimize implementation of the PrEP shared decision-making tool. As noted, our team also developed an HCP PrEP information resource and data gathered through formative work and piloting of the PrEP shared decision-making tool, including from HCPs who viewed the HCP PrEP information resource in preparation for mock clinical encounters, would inform development of a resource to support HCP readiness for using the PrEP shared decision-making tool. Healthcare providers also suggested the value of a brief training around the PrEP shared decision-making tool and, again, findings from this research could inform the development of training modules and other potential supports. People who could benefit from PrEP also had ideas about additional information they might want to access as they learn about PrEP choice, and this could also be organized and presented in supplementary PrEP literacy materials for people who could benefit from PrEP.

Mechanics of dissemination and the dynamics of how the PrEP shared decision-making tool is used will also be important to assess. For example, it would be useful to evaluate how targeted and intentional dissemination of the PrEP shared decision-making tool and any additional supplementary resources support its uptake. Also important is evaluating the impact of introducing the PrEP shared decision-making tool and any supplementary materials and supports in different settings. For example, it would be instructive to look at how introduction of the PrEP shared decision-making tool and supplementary materials supports increased awareness of PrEP modalities within specific clinical care environments (e.g., HIV/STI care, reproductive health, primary care, etc.), especially given current recommendations that all sexually active individuals be offered PrEP [62]. Finally, as LAI PrEP becomes available in global contexts, it will be important to consider if and how to adapt a PrEP shared decision-making tool to different cultural and geographic settings including, for example, those where injections are viewed with greater acceptance or where injection drug use is prevalent. Proposed adaptations and evaluations of the PrEP shared decision-making tool should be guided by models such as the Ottawa Decision Support Framework [63] which guides researchers in the evaluation of decisional needs, decision support and decisional outcomes as well as the International Patient Decision Aid Standards (IPDAS) which offers checklists for evaluating the quality of decision aids [64]. Additionally, as new PrEP modalities and options continue to evolve [61,65], these may be able to be incorporated into the shared decision-making tool developed here for further evolution and evaluation.

We developed and piloted one of the first PrEP shared decision-making tools to be used with diverse people who could benefit from PrEP and their HCPs across clinic types and settings. The PrEP shared decision-making tool demonstrated strong acceptability and streamlined critical conversations regarding PrEP choices. The tool offers an acceptable and promising mechanism for addressing disparities in awareness of PrEP options and discomfort with discussing PrEP and disclosing HIV risk which can foster disparities in PrEP access and uptake. Testing the effectiveness of the PrEP shared decision-making tool on PrEP awareness and access in a larger-scale evaluation is highly warranted as the field seeks to diminish ongoing inequities in PrEP uptake across gender, racial and ethnic groups or regions, in the U.S. [59,66–69] and globally [49,70].

## Abbreviations

FDA: Food and Drug administration
HCP: healthcare provider
IDI: in-depth interview
LAI: long-acting injectable
PrEP: pre-exposure prophylaxis
STI: sexually transmitted infection,
US: United States
